# Comparative Effectiveness of Lifestyle Versus Pharmacologic Therapy in Non-alcoholic Fatty Liver Disease (NAFLD): A 12-Month Multicenter Observational Study

**DOI:** 10.7759/cureus.92865

**Published:** 2025-09-21

**Authors:** Zubair Ahmad, Muhammad Hamza Ghufran, Suleman Khan, Zeeshan Muzaffar, Qazi Muhammad Safwan, Muhammad Nouman Shuja, Hamza Usman, Usman Khan, Saeed Ullah, Syed Ali Abdullah Jan, Naqeeb Ullah

**Affiliations:** 1 Department of Internal Medicine, Hayatabad Medical Complex, Peshawar, PAK; 2 Department of Medicine, Lady Reading Hospital, Peshawar, PAK; 3 Department of Pathology, Northwest School of Medicine, Peshawar, PAK; 4 Department of Neurology, Hayatabad Medical Complex, Peshawar, PAK; 5 Department of Surgical Oncology, Shaukat Khanum Memorial Cancer Hospital and Research Centre, Peshawar, PAK; 6 Department of Internal Medicine, Saidu Group of Teaching Hospitals, Saidu Sharif, PAK; 7 Department of Internal Medicine, Mardan Medical Complex (MMC), Mardan, PAK; 8 Department of Internal Medicine, Lady Reading Hospital, Peshawar, PAK

**Keywords:** fibroscan, lifestyle intervention, liver enzymes, metabolic syndrome, nafld, pharmacological therapy

## Abstract

Background: Non-alcoholic fatty liver disease (NAFLD) is a growing global health concern linked to metabolic syndrome (MetS) and insulin resistance.

Objective: The objective of the study is to compare the effectiveness of lifestyle interventions versus pharmacological therapy in the management of NAFLD across multiple healthcare centers using a real-world observational cohort design.

Methodology: This multicentric observational cohort study was conducted from January 2023 to December 2024 at the Lady Reading Hospital (LRH), Peshawar, and Hayatabad Medical Complex (HMC), Peshawar. The 12-month follow-up was completed by 367 (90.84%) of the 404 patients who were recruited. Participants were split into two groups: one for pharmaceutical treatment (n = 179) and another for lifestyle intervention (n = 188). At baseline and after a year, clinical, biochemical, and radiological data were evaluated. Statistical Package for the Social Sciences (SPSS) version 26 (IBM Corp., Armonk, NY, US) was used for data analysis.

Results: The lifestyle group showed significantly greater reductions in weight (-4.52 ± 2.10 kg vs. -1.65 ± 1.44 kg; p < 0.001), body mass index (BMI) (-1.43 ± 0.65 vs. -0.52 ± 0.48 kg/m²; p < 0.001), alanine aminotransferase (ALT) (-18.77 ± 10.84 vs. -15.23 ± 9.45 U/L; p = 0.004), and FibroScan (transient elastography) scores (-1.98 ± 0.84 vs. -1.47 ± 0.78 kPa; p < 0.001) compared to the pharmacological group. ALT reduction > 30% was observed in 112 (59.57%) lifestyle patients versus 89 (49.72%) pharmacological patients (p = 0.048), and BMI reduction ≥ 5% in 106 (56.38%) vs. 41 (22.91%), respectively (p < 0.001).

Conclusion: Lifestyle intervention is more effective than pharmacological therapy in improving metabolic and liver-related outcomes in NAFLD patients.

## Introduction

The global obesity, insulin resistance, type 2 diabetes mellitus, and metabolic syndrome epidemics are intimately associated with non-alcoholic fatty liver disease (NAFLD), which has become the most common chronic liver disease globally [1,2]. NAFLD, which includes a variety of liver diseases from basic steatosis to non-alcoholic steatohepatitis, fibrosis, cirrhosis, and even hepatocellular cancer, is defined by the excessive buildup of hepatic fat without substantial alcohol use [3]. Early on, it is often asymptomatic, which postpones diagnosis and increases long-term morbidity and healthcare costs [4].

Modifiable lifestyle variables, namely, poor eating habits, physical inactivity, and sedentary behavior, have been linked more and more in recent decades to the etiology of NAFLD [5]. The therapeutic care of NAFLD has changed as a result of these discoveries, moving toward long-term risk reduction and preventative measures [6]. Pharmaceutical treatments have drawn attention, especially in moderate to severe instances or when lifestyle adherence is insufficient, even if lifestyle changes, such as weight reduction via food restriction and frequent exercise, remain the mainstay of initial NAFLD care [7].

Numerous pharmacological treatments, including sodium-glucose cotransporter-2 (SGLT2) inhibitors, pioglitazone, vitamin E, and glucagon-like peptide-1 (GLP-1) receptor agonists, have shown varied degrees of histological and metabolic improvement in individuals with NAFLD [8]. However, there is still a lack of research on the relative efficacy of lifestyle modifications vs. pharmaceutical therapy, especially in multi-institutional real-world settings [9]. Furthermore, the generalizability of trial-based recommendations to wider clinical practice is further complicated by inter-individual heterogeneity in treatment response and adherence patterns [10].

A comparison of the two treatment modalities is essential due to the rising prevalence of NAFLD and its close correlation with metabolic and lifestyle risk factors. In environments with low resources, where many patients may not find long-term pharmaceutical therapy viable or sustainable, this becomes even more crucial. Knowing which modality performs better in a range of patient demographics can help develop more individualized and economical treatment plans.

Research objective

The objective of the research is to compare the effectiveness of lifestyle interventions versus pharmacological therapy in the management of NAFLD across multiple healthcare centers using a real-world observational cohort design.

## Materials and methods

Study design and setting

This multicentric prospective observational cohort study was conducted from January 2023 to December 2024 at the Lady Reading Hospital (LRH), Peshawar, and the Hayatabad Medical Complex (HMC), Peshawar. Patients with a confirmed diagnosis of NAFLD were enrolled and followed for 12 months to compare the effectiveness of lifestyle interventions versus pharmacological therapy. The study has been reported in accordance with the STROBE (Strengthening the Reporting of Observational Studies in Epidemiology) guidelines.

Inclusion and exclusion criteria

Adults between 18 and 65 years of age with a diagnosis of NAFLD, confirmed through clinical evaluation, biochemical investigations, and radiological assessment using ultrasonography, were eligible for inclusion. Only those able to provide written informed consent and commit to either pharmaceutical therapy or lifestyle intervention were enrolled. Patients with a history of heavy alcohol consumption (more than 20 g/day for women and more than 30 g/day for men) were excluded. Additional exclusion criteria included the presence of chronic liver diseases such as viral hepatitis, autoimmune hepatitis, or hemochromatosis; pregnancy or lactation; current use of hepatotoxic medications; concurrent participation in another clinical trial; or inability to comply with the 12-month follow-up protocol.

Intervention groups

Patients assigned to the pharmaceutical therapy group received standard pharmacological treatment tailored according to physician assessment, comorbidities, and drug availability. The therapeutic options included SGLT2 inhibitors, GLP-1 receptor agonists, pioglitazone, and vitamin E supplementation, all prescribed in accordance with international guidelines for the management of NAFLD. To improve reproducibility, the most frequently prescribed agents were dapagliflozin (10 mg once daily), liraglutide (up to 1.8 mg once daily), pioglitazone (15-30 mg once daily), and vitamin E (800 IU once daily). Final drug selection and dose adjustments were made at the discretion of the treating physician based on comorbid conditions and patient tolerance. Adherence was monitored at each follow-up visit.

Participants in the lifestyle intervention group were provided with structured guidance by physicians and dietitians. The program involved individualized dietary modification with a calorie-restricted, balanced diet (approximately 30% fat, 15%-20% protein, and 50%-55% carbohydrates). Patients were advised to engage in a minimum of 150 minutes of moderate-intensity aerobic exercise per week, complemented by two weekly sessions of strength training. Counseling sessions also addressed sleep hygiene, stress reduction, and behavioral strategies to promote long-term adherence. Compliance with the lifestyle program was evaluated using patient diaries and reinforced during follow-up consultations.

Sample size

The sample size was calculated using Cochran’s formula, assuming a 95% confidence interval (Z = 1.96), a 5% margin of error, and an estimated population proportion of 0.30 based on the reported regional prevalence of NAFLD. This yielded a minimum required sample size of 323. To accommodate an anticipated 20% attrition rate, the final sample size was increased to 404 patients, equally divided between the pharmaceutical and lifestyle groups. By the end of the 12-month follow-up, 367 patients (90.84%) had completed the study, including 188 in the lifestyle group and 179 in the pharmaceutical group. A total of 37 patients (9.16%) were lost to follow-up due to relocation, protocol non-compliance, withdrawal of consent, or loss of contact.

Data collection

Participants were recruited sequentially based on physician recommendation and patient preference. Patient preference was documented through structured interviews during the initial consultation, where patients were asked to indicate whether they would be more comfortable pursuing lifestyle modification or pharmacological therapy or were open to either approach. This record was entered into the case report form (CRF) to ensure transparency in allocation. At baseline, demographic information, comorbidities, and clinical data were recorded, along with biochemical parameters such as body mass index (BMI), glycated hemoglobin (HbA1c), lipid profile, and liver function tests including alanine aminotransferase (ALT) and aspartate aminotransferase (AST).

Radiological evaluation was performed using abdominal ultrasonography with a Mindray DC-70 X-Insight system (Mindray, Shenzhen, China). Hepatic steatosis was graded using standard sonographic criteria, including echogenicity relative to the renal cortex, clarity of vascular interfaces, and visualization of the diaphragm. Additionally, FibroScan (Echosens, Paris, France) was employed to measure liver stiffness, with results reported in kilopascals (kPa). All imaging was performed by experienced radiologists, and inter-observer variability was minimized through independent double readings with adjudication by a third radiologist when necessary. Follow-up evaluations were conducted at three, six, and 12 months, during which weight, BMI, metabolic markers, liver enzyme levels, and imaging findings were reassessed.

Statistical analysis

All statistical analyses were performed using the Statistical Package for the Social Sciences (SPSS) version 26 (IBM Corp., Armonk, NY, US). Continuous variables were expressed as mean ± standard deviation (SD) and analyzed using independent t-tests to compare between groups and paired t-tests to assess within-group changes over time. Categorical variables were compared using the Chi-squared test or Fisher’s exact test, as appropriate. A p-value of less than 0.05 was considered statistically significant.

Ethical approval

The study was conducted in accordance with the principles outlined in the Declaration of Helsinki. Ethical clearance was obtained from the Institutional Review Board (IRB) of LRH, Peshawar, under approval number 568/LRH/MTI (dated December 14, 2022). Written informed consent was obtained from all participants prior to enrollment.

## Results

A total of 367 participants completed the study, including 188 patients in the lifestyle intervention group and 179 patients in the pharmacological therapy group. Table 1 presents baseline characteristics, showing no statistically significant differences between the two groups. The mean age was 45.12 ± 10.23 years in the lifestyle group and 44.87 ± 9.89 years in the pharmacological group (p = 0.72). Men comprised 53.72% (n = 101) of the lifestyle group and 53.07% (n = 95) of the pharmacological group (p = 0.91). The mean BMI was 31.48 ± 3.52 kg/m² vs. 31.22 ± 3.66 kg/m² (p = 0.38), and the mean HbA1c was 6.78% ± 0.62% vs. 6.81% ± 0.69% (p = 0.61). NAFLD with fibrosis stage ≥ F2 was present in 34.57% (n = 65) of the lifestyle group and 37.43% (n = 67) of the pharmacological group (p = 0.59).

**Table 1:** Baseline demographic and clinical characteristics Independent t-test (t) was applied for continuous variables, and Chi square (χ²) for categorical variables. BMI: body mass index; HbA1c: hemoglobin A1c; ALT: alanine aminotransferase; AST: aspartate aminotransferase; T2DM: type 2 diabetes mellitus; NAFLD: non-alcoholic fatty liver disease.

Variable	Lifestyle group (n = 188)	Pharmacological group (n = 179)	Test statistic (df)	p-value
Age (years)	45.12 ± 10.23	44.87 ± 9.89	t(365) = 0.36	0.72
Male, n (%)	101 (53.72)	95 (53.07)	χ²(1) = 0.01	0.91
Female, n (%)	87 (46.28)	84 (46.93)		
BMI (kg/m²)	31.48 ± 3.52	31.22 ± 3.66	t(365) = 0.87	0.38
HbA1c (%)	6.78 ± 0.62	6.81 ± 0.69	t(365) = 0.51	0.61
ALT (U/L)	72.34 ± 15.87	73.11 ± 16.24	t(365) = 0.61	0.54
AST (U/L)	65.22 ± 13.45	64.76 ± 14.09	t(365) = 0.37	0.71
Dyslipidemia, n (%)	108 (57.45)	104 (58.10)	χ²(1) = 0.02	0.89
Hypertension, n (%)	76 (40.43)	71 (39.66)	χ²(1) = 0.02	0.88
T2DM, n (%)	84 (44.68)	82 (45.81)	χ²(1) = 0.04	0.83
NAFLD with fibrosis (≥F2), n (%)	65 (34.57)	67 (37.43)	χ²(1) = 0.29	0.59

Table 2 shows the between-group comparisons after 12 months. The lifestyle group had significantly greater weight loss (-4.52 ± 2.10 kg vs. -1.65 ± 1.44 kg, p < 0.001), BMI reduction (-1.43 ± 0.65 kg/m² vs. -0.52 ± 0.48 kg/m², p < 0.001), ALT reduction (-18.77 ± 10.84 U/L vs. -15.23 ± 9.45 U/L, p = 0.004), and AST reduction (-16.53 ± 8.91 U/L vs. -13.45 ± 7.32 U/L, p = 0.002). Improvements in FibroScan scores were also greater in the lifestyle group (-1.98 ± 0.84 kPa vs. -1.47 ± 0.78 kPa, p < 0.001).

**Table 2:** Clinical and biochemical changes at 12 months Independent t-test (t) was used for between-group comparisons at each time point. BMI: body mass index; HbA1c: hemoglobin A1c; ALT: alanine aminotransferase; AST: aspartate aminotransferase; LDL-C: low-density lipoprotein cholesterol.

Parameter	Time point	Lifestyle group (n = 188)	Pharmacological group (n = 179)	Test statistic (df)	p-value
Weight change (kg)	3 mo	-1.58 ± 0.92	-0.73 ± 0.65	t(365) = 10.87	<0.001
	6 mo	-3.12 ± 1.41	-1.10 ± 0.93	t(365) = 15.23	<0.001
	12 mo	-4.52 ± 2.10	-1.65 ± 1.44	t(365) = 16.91	<0.001
BMI change (kg/m²)	3 mo	-0.58 ± 0.34	-0.21 ± 0.19	t(365) = 11.26	<0.001
	6 mo	-0.98 ± 0.52	-0.34 ± 0.29	t(365) = 14.72	<0.001
	12 mo	-1.43 ± 0.65	-0.52 ± 0.48	t(365) = 17.02	<0.001
HbA1c change (%)	3 mo	-0.15 ± 0.11	-0.13 ± 0.10	t(365) = 1.47	0.14
	6 mo	-0.28 ± 0.19	-0.25 ± 0.18	t(365) = 1.02	0.31
	12 mo	-0.42 ± 0.28	-0.48 ± 0.33	t(365) = 1.88	0.06
ALT change (U/L)	3 mo	-6.24 ± 4.31	-4.11 ± 3.76	t(365) = 4.62	<0.001
	6 mo	-12.56 ± 7.22	-9.12 ± 6.45	t(365) = 5.19	<0.001
	12 mo	-18.77 ± 10.84	-15.23 ± 9.45	t(365) = 6.72	0.004
AST change (U/L)	3 mo	-5.33 ± 3.76	-3.74 ± 3.22	t(365) = 4.28	<0.001
	6 mo	-11.12 ± 6.23	-8.31 ± 5.87	t(365) = 5.01	<0.001
	12 mo	-16.53 ± 8.91	-13.45 ± 7.32	t(365) = 6.38	0.002
Triglyceride change (mg/dL)	3 mo	-10.56 ± 7.21	-8.42 ± 6.98	t(365) = 3.25	0.001
	6 mo	-22.31 ± 12.16	-18.03 ± 11.09	t(365) = 4.02	<0.001
	12 mo	-32.11 ± 15.09	-28.76 ± 14.63	t(365) = 5.22	0.03
LDL-C change (mg/dL)	3 mo	-7.44 ± 5.76	-8.21 ± 5.44	t(365) = 1.13	0.26
	6 mo	-15.33 ± 9.17	-16.02 ± 8.92	t(365) = 0.87	0.38
	12 mo	-21.44 ± 11.23	-23.12 ± 10.88	t(365) = 1.58	0.19
FibroScan score change (kPa)	3 mo	-0.72 ± 0.41	-0.48 ± 0.39	t(365) = 6.15	<0.001
	6 mo	-1.28 ± 0.63	-0.92 ± 0.59	t(365) = 7.04	<0.001
	12 mo	-1.98 ± 0.84	-1.47 ± 0.78	t(365) = 8.21	<0.001

Table 3 illustrates within-group changes. In the lifestyle group, ALT decreased from 72.34 ± 15.87 U/L to 53.57 ± 13.94 U/L, AST from 65.22 ± 13.45 U/L to 48.69 ± 11.21 U/L, BMI from 31.48 to 30.05 kg/m², HbA1c from 6.78% to 6.36%, and FibroScan score from 6.42 to 4.44 kPa (p < 0.001 for all). In the pharmacological group, ALT dropped from 73.11 ± 16.24 to 57.88 ± 13.76 U/L, AST from 64.76 ± 14.09 to 51.31 ± 11.94 U/L, BMI from 31.22 to 30.70 kg/m², HbA1c from 6.81% to 6.33%, and FibroScan from 6.38 to 4.91 kPa (p < 0.001 for all).

**Table 3:** Within-group changes in biochemical and clinical parameters from baseline to 12-month follow-up Paired t-test (t) was used for within-group comparisons across time points. ALT: alanine aminotransferase; AST: aspartate aminotransferase; BMI: body mass index; HbA1c: hemoglobin A1c; SD: standard deviation.

Parameter	Group	Baseline mean ± SD	3 mo	6 mo	12 mo	Test statistic (df)	p-value
ALT (U/L)	Lifestyle	72.34 ± 15.87	66.10 ± 14.12	59.78 ± 13.45	53.57 ± 13.94	t(187) = 12.14	<0.001
	Pharma	73.11 ± 16.24	68.77 ± 15.32	62.34 ± 14.21	57.88 ± 13.76	t(178) = 10.47	<0.001
AST (U/L)	Lifestyle	65.22 ± 13.45	60.14 ± 12.89	54.12 ± 11.78	48.69 ± 11.21	t(187) = 11.86	<0.001
	Pharma	64.76 ± 14.09	60.55 ± 13.66	55.11 ± 12.33	51.31 ± 11.94	t(178) = 10.28	<0.001
BMI (kg/m²)	Lifestyle	31.48 ± 3.52	30.90 ± 3.40	30.42 ± 3.32	30.05 ± 3.24	t(187) = 14.92	<0.001
	Pharma	31.22 ± 3.66	30.98 ± 3.58	30.82 ± 3.52	30.70 ± 3.45	t(178) = 7.21	<0.001
HbA1c (%)	Lifestyle	6.78 ± 0.62	6.61 ± 0.56	6.47 ± 0.51	6.36 ± 0.48	t(187) = 9.38	<0.001
	Pharma	6.81 ± 0.69	6.65 ± 0.63	6.49 ± 0.57	6.33 ± 0.51	t(178) = 8.72	<0.001
FibroScan (kPa)	Lifestyle	6.42 ± 1.23	5.70 ± 1.15	5.04 ± 1.14	4.44 ± 1.12	t(187) = 13.27	<0.001
	Pharma	6.38 ± 1.15	5.91 ± 1.13	5.36 ± 1.10	4.91 ± 1.08	t(178) = 11.92	<0.001

**Figure 1:**
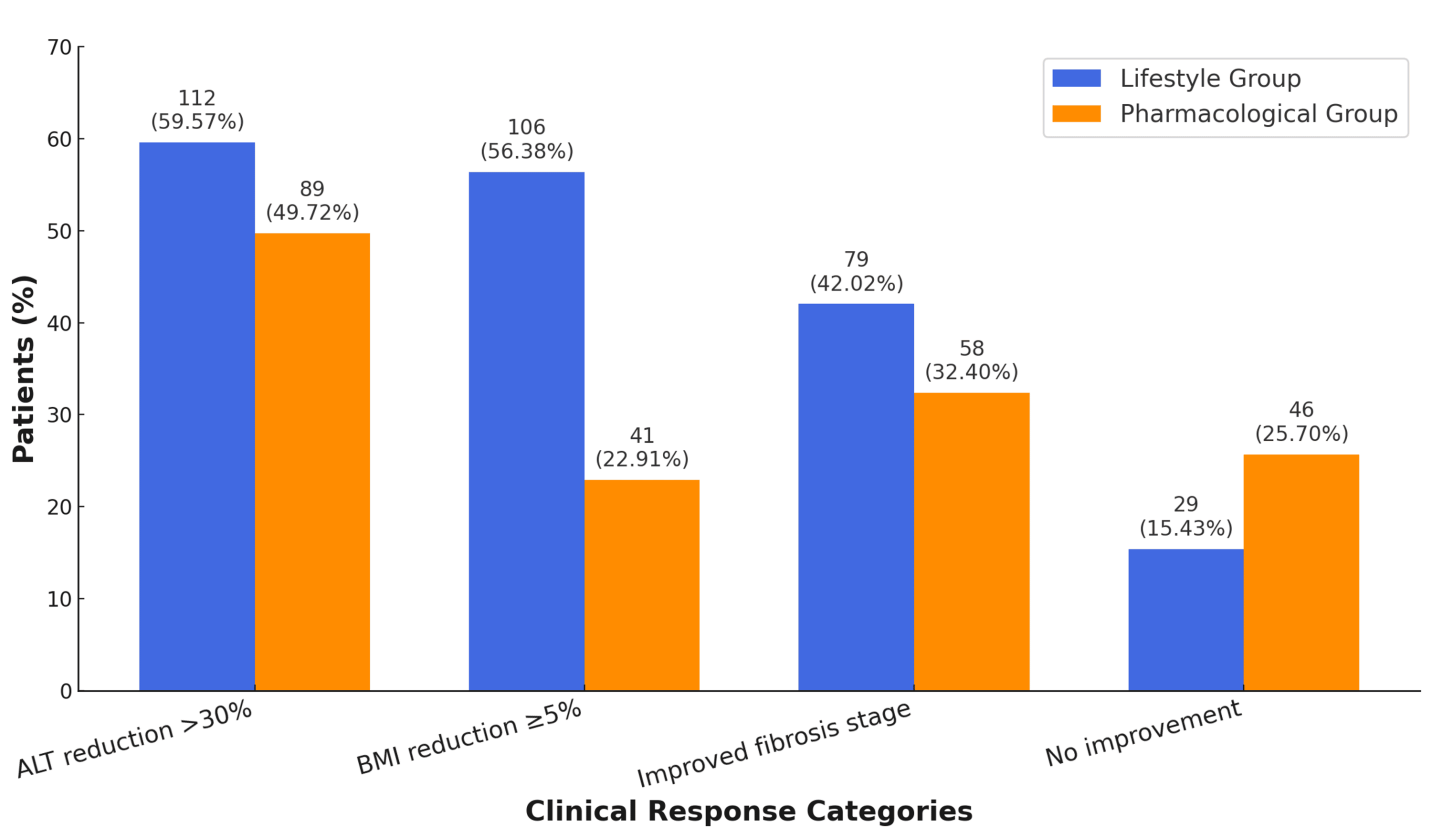
Clinical response categories at 12-month follow-up Bars represent the proportion of patients achieving categorical treatment responses: alanine aminotransferase (ALT) reduction > 30%, body mass index (BMI) reduction ≥ 5%, and improvement in fibrosis stage. “No improvement” indicates patients without meaningful clinical or biochemical improvement at follow-up. The Y-axis represents percentages (%). Counts (n) are displayed on top of bars. Group comparisons were performed using the Chi-squared test (χ²).

Figure 1 shows categorical treatment responses at 12 months. ALT reduction > 30% was observed in 112 patients (59.57%) in the lifestyle group versus 89 patients (49.72%) in the pharmacological group (p = 0.048). BMI reduction ≥ 5% occurred in 106 patients (56.38%) in the lifestyle group versus 41 patients (22.91%) in the pharmacological group (p < 0.001). Improvement in fibrosis stage was noted in 79 patients (42.02%) in the lifestyle group and 58 patients (32.40%) in the pharmacological group (p = 0.049). Notably, no improvement was observed in 29 patients (15.43%) in the lifestyle group compared to 46 patients (25.70%) in the pharmacological group (p = 0.017).

## Discussion

Our multicentric observational cohort study's results provide strong proof that, during a 12-month follow-up period, lifestyle modifications are superior to pharmaceutical treatment for the management of NAFLD. Compared to the pharmaceutical group (−1.65 ± 1.44 kg and −0.52 ± 0.48 kg/m², respectively), patients in the lifestyle group in our research showed considerably larger decreases in body weight (−4.52 ± 2.10 kg) and BMI (−1.43 ± 0.65 kg/m²; p < 0.001). These results are in line with the earlier study that showed that dietary restriction and increased physical activity, among other lifestyle changes, led to a 5%-10% decrease in body weight, which in turn improved the histology of NAFLD patients [11].

Significant biochemical improvements in the lifestyle group are also shown by our results. In contrast to the pharmaceutical group (−15.23 ± 9.45 U/L and −13.45 ± 7.32 U/L; p = 0.004 and p = 0.002, respectively), the lifestyle group saw more significant decreases in ALT and AST (−18.77 ± 10.84 U/L and −16.53 ± 8.91 U/L). These findings are consistent with earlier research that showed that organized lifestyle treatments, such as exercise, substantially lowered ALT levels in individuals with NAFLD and improved biochemical markers when paired with medication [9].

FibroScan's measurement of fibrosis improvement was one of our study's noteworthy results. The pharmaceutical group's mean kilopascal decrease was −1.47 ± 0.78, but the lifestyle group's was −1.98 ± 0.84 (p < 0.001). This is consistent with earlier research showing that modest weight reduction brought on by lifestyle modifications significantly improved liver biopsy steatosis and fibrosis scores [12]. Although there were notable decreases in HbA1c in both groups, the difference between them was not statistically significant (p = 0.06) (−0.42% in lifestyle vs. −0.48% in pharmaceutical). This implies that, in line with other observational research, lifestyle modifications provide wider advantages across a variety of metabolic and hepatic markers, even when both approaches enhance glycemic control [13]. Furthermore, categorical results showed that 56.38% (n = 106) of the lifestyle group and only 22.91% (n = 41) of the pharmaceutical group achieved a BMI decrease of ≥5% (p < 0.001). The prior research, which emphasized the challenge of attaining clinically significant weight reduction with pharmacologic treatments alone without behavioral assistance, found similar proportions [14,15].

This study's multicenter, real-world design and emphasis on the Pakistani population-a group underrepresented in NAFLD research-make it significant. The research improves the external validity of its conclusions by comparing two widely utilized therapy modalities in standard clinical settings. Additionally, it emphasizes the durability and cost-effectiveness of lifestyle changes, especially in healthcare systems with limited resources, providing useful information for doctors and policymakers looking to reduce the rising prevalence of NAFLD.

Strengths and limitations

A substantial sample size (n = 367), a multicentric design combining two tertiary care facilities, and a real-world observational cohort approach are some of this study's qualities that improve the results' applicability to standard clinical practice. A thorough comparison of the clinical, biochemical, and radiological results between the pharmaceutical and lifestyle groups was made possible by the prospective nature of the data collection and the organized follow-up at three, six, and 12 months. Nonetheless, it is necessary to recognize certain restrictions. Due to the lack of randomization in patient allocation, selection bias based on patient or physician choice may have occurred. Furthermore, reporting bias could have resulted from the use of self-reported adherence to lifestyle changes. Despite using FibroScan as a non-invasive surrogate, the histological confirmation of improvement is limited by the absence of liver biopsy data. Finally, the research did not take into consideration the combination of pharmaceutical and lifestyle therapy, which may be more representative of hybrid treatment approaches used in the real world.

## Conclusions

This multicenter observational cohort research shows that over a 12-month period, lifestyle interventions improve important clinical and biochemical indicators in patients with NAFLD more effectively than pharmaceutical treatment. FibroScan measurements of body weight, BMI, liver enzyme levels, and liver stiffness were all lower in patients who changed their lifestyles. Meaningful clinical responses, such as a ≥5% decrease in BMI and a >30% improvement in ALT levels, were also attained by a noticeably larger percentage. These results highlight the need to advocate for organized lifestyle treatments as the first line of treatment for NAFLD, especially in low-resource environments where long-term medication may not be practical or sustainable.

## References

[1] Mitrovic B, Gluvic ZM, Obradovic M, Radunovic M, Rizzo M, Banach M, Isenovic ER (2023). Non-alcoholic fatty liver disease, metabolic syndrome, and type 2 diabetes mellitus: where do we stand today?. Arch Med Sci.

[2] Stefan N, Cusi K (2022). A global view of the interplay between non-alcoholic fatty liver disease and diabetes. Lancet Diabetes Endocrinol.

[3] Grander C, Grabherr F, Tilg H (2023). Non-alcoholic fatty liver disease: pathophysiological concepts and treatment options. Cardiovasc Res.

[4] Huang TD, Behary J, Zekry A (2020). Non-alcoholic fatty liver disease: a review of epidemiology, risk factors, diagnosis and management. Intern Med J.

[5] Al-Dayyat HM, Rayyan YM, Tayyem RF (2018). Non-alcoholic fatty liver disease and associated dietary and lifestyle risk factors. Diabetes Metab Syndr.

[6] Del Ben M, Polimeni L, Baratta F, Pastori D, Loffredo L, Angelico F (2014). Modern approach to the clinical management of non-alcoholic fatty liver disease. World J Gastroenterol.

[7] Yoo KD, Jun DW (2014). Nonalcoholic fatty liver disease: lifestyle modification. Korean J Med.

[8] Gu Y, Sun L, He Y (2023). Comparative efficacy of glucagon-like peptide 1 (GLP-1) receptor agonists, pioglitazone and vitamin E for liver histology among patients with nonalcoholic fatty liver disease: systematic review and pilot network meta-analysis of randomized controlled trials. Expert Rev Gastroenterol Hepatol.

[9] Kandel A, Pant P, Todi S, Kc S, Pandey S (2024). Effect of exercise and pharmacotherapy on non-alcoholic fatty liver disease. SAGE Open Med.

[10] Mantovani A, Dalbeni A (2021). Treatments for NAFLD: state of art. Int J Mol Sci.

[11] Vilar-Gomez E, Martinez-Perez Y, Calzadilla-Bertot L (2015). Weight loss through lifestyle modification significantly reduces features of nonalcoholic steatohepatitis. Gastroenterology.

[12] Dixon JB, Bhathal PS, Hughes NR, O'Brien PE (2004). Nonalcoholic fatty liver disease: improvement in liver histological analysis with weight loss. Hepatology.

[13] Adams LA, Angulo P (2006). Treatment of non-alcoholic fatty liver disease. Postgrad Med J.

[14] Bellentani S, Dalle Grave R, Suppini A, Marchesini G (2008). Behavior therapy for nonalcoholic fatty liver disease: the need for a multidisciplinary approach. Hepatology.

[15] Petroni ML, Brodosi L, Bugianesi E, Marchesini G (2021). Management of non-alcoholic fatty liver disease. BMJ.

